# Erythrocyte-Associated Proteins Underlie the Reported Oxidative-Stress Enrichment in Plasma Proteomics of Obstructive Sleep Apnoea: A Secondary Quality Marker Analysis of Public Datasets

**DOI:** 10.3390/jcm15208081

**Published:** 2026-10-17

**Authors:** Seyma Yasar, Fatma Hilal Yagin, Busra Nur Findik, Sarah A. Alzakari, Amal K. Alkhalifa, Fahaid Al-Hashem, Abedelmalek Kalefh Tabnjh

**Affiliations:** 1 Department of Biostatistics and Medical Informatics, Faculty of Medicine, Inonu University, Malatya 44280, Türkiye; seyma.yasar@inonu.edu.tr; 2 Department of Biostatistics, and Medical Informatics, Faculty of Medicine, Balikesir University, Balikesir 10145, Türkiye; 3 Department of Therapy and Rehabilitation, Kozaklı Vocational School, Nevşehir Hacı Bektaş Veli University, Nevşehir 50600, Türkiye; busranurfindik@nevsehir.edu.tr; 4 Department of Computer Sciences, College of Computer and Information Sciences, Princess Nourah bint Abdulrahman University, P.O. Box 84428, Riyadh 11671, Saudi Arabia; saalzakari@pnu.edu.sa (S.A.A.); akalkalifh@pnu.edu.sa (A.K.A.); 5 Department of Physiology, College of Medicine, King Khalid University, Abha 61421, Saudi Arabia; fahaid999@yahoo.com; 6 Department of Cariology, Institute of Odontology, The Sahlgrenska Academy, University of Gothenburg, 41390 Gothenburg, Sweden; 7 Department of Applied Dental Sciences, Faculty of Applied Medical Sciences, Jordan University of Science and Technology, Irbid 22110, Jordan; 8 Department of Pathology, Saveetha Medical College and Hospital, Saveetha Institute of Medical and Technical Sciences, Chennai 602 105, Tamil Nadu, India

**Keywords:** plasma proteomics, obstructive sleep apnoea, disease severity, preanalytical variability, erythrocyte-associated proteins, biomarker validation, quality control, data reuse

## Abstract

**Background:** Erythrocyte proteins released into plasma carry oxidation-related annotations and can be read as evidence of oxidative stress. We examined whether this applies to an oxidative-stress enrichment reported in a plasma proteomic study of obstructive sleep apnoea (OSA). **Methods:** In a secondary analysis of public data (PXD073409; men; 15 non-OSA, 15 mild OSA, 15 severe OSA; 422 proteins), erythrocyte, platelet and coagulation quality marker indices were computed from the panels of Geyer et al.; the erythrocyte panel was only partly quantified (15 of 29 entries). The original differential and over-representation analyses were repeated with panel members excluded, with adjustment for the erythrocyte index, and with leave-one-protein-out indices. A second public dataset (PXD069503; 15 severe OSA, 15 mild-to-moderate OSA, 15 controls) was used for directional comparison. Clinical covariates were not available. **Results:** The erythrocyte index was higher in mild (ratio of geometric means 1.74, 95% CI 1.04 to 2.80) and severe OSA (2.43, 1.68 to 3.47) than in non-OSA participants. Six of the 13 proteins that are increased in severe OSA were erythrocyte panel members, and all nine enriched terms consisted of panel members only, including hydrogen peroxide catabolic process (HBA1, HBB, HBD, PRDX2). After adjustment, PRDX2 was accounted for by erythrocyte content, whereas the haemoglobin chains remained elevated and carried seven of the terms. The index alone separated severe- from non-OSA participants (AUROC 0.858); the data could not show whether all 422 proteins (AUROC 0.969) add to it (difference +0.111, 95% CI −0.009 to +0.268). In the second dataset the index was higher in severe OSA than in controls (1.55, 1.06 to 2.35). **Conclusions:** The reported oxidative-stress enrichment rests on erythrocyte-associated proteins and, on these data, is not evidence of altered oxidative activity in plasma. Whether this content arises before or after venepuncture cannot be determined. Quality marker indices should be reported alongside plasma proteomic biomarker results.

## 1. Introduction

Obstructive sleep apnoea (OSA) is common and largely undiagnosed, and its diagnosis depends on polysomnography; this has motivated interest in blood biomarkers, both for screening and for characterising the systemic consequences of the disease [1,2,3].

Blood plasma is accordingly the most frequently interrogated matrix in biomarker research. Mass spectrometry now quantifies thousands of plasma proteins per run, and depth continues to increase with immunoaffinity depletion, bead-based, and nanoparticle-enrichment strategies [4,5,6]. Depth, however, is not what determines whether a discovered signal is real. Plasma is separated from a cellular compartment far more concentrated in protein than plasma itself, and even minor carry-over of erythrocyte or platelet content shifts the measured proteome systematically rather than randomly [7].

This vulnerability is monitored routinely in laboratory medicine but is reported inconsistently in proteomics. A recent systematic review of preanalytical variability in plasma proteomics found that most eligible studies detected significant proteome changes attributable to handling alone, with time to processing and temperature most often implicated [8]. Comparable effects have been demonstrated for anticoagulant choice, tube type, centrifugation stringency, extracellular vesicle content and standard operating procedure variation [9,10,11,12,13,14]; for enrichment chemistry, where cellular carry-over can inflate apparent protein counts substantially [5,6,15]; and for affinity platforms, where a large fraction of Olink probes was significantly affected by in vitro haemolysis [16]. Plasma proteome composition can even shift measurably within the time frame of a single clinical event [17].

Geyer et al. addressed the problem quantitatively [7]. They derived deep reference proteomes of erythrocytes, platelets and platelet-free plasma from 20 healthy donors and distilled them into three quality marker panels of approximately 30 proteins each. Serial spike-in dilutions with parallel cell counting established that the resulting quality marker indices scale linearly with contaminating cell number (R = 0.98 for erythrocytes), making them calibrated measurements rather than heuristics. In a survey of 210 published biomarker studies, 54% reported at least one quality panel protein as a candidate biomarker. The authors further warned that erythrocyte leakage proteins—haemoglobin subunits, carbonic anhydrases, peroxiredoxin 2, superoxide dismutase and aldolase A—carry oxidation-related annotations and may therefore be misread as evidence of oxidative pathway activation, illustrating the risk with a study that had linked plasma proteome alterations in type 1 diabetes to oxidative stress.

OSA is a setting in which this matters. Intermittent hypoxia and reoxygenation are held to generate reactive oxygen species, and oxidative stress is an established component of the accepted account of OSA-related end-organ injury [18,19]. A proteomic finding of increased antioxidant or oxidative-stress proteins in severe OSA therefore has strong prior plausibility, and may for that reason receive less scrutiny as a possible artefact.

The picture is further complicated by genuine erythrocyte involvement in OSA. Red blood cell proteomic profiling has shown severity-dependent changes in glycolytic and pentose phosphate pathway enzymes [20]; a disrupted erythrocyte S1P-eNOS axis with excessive oxidative stress has been described as an early pathogenic mechanism [21]; haemoglobin concentration and red cell distribution width are associated with severity and outcome in OSA cohorts [22,23,24,25]; and exhaled carbon monoxide has been examined as a haemolysis surrogate in OSA patients [26]. Distinguishing an erythrocyte-derived signal that originates in the collection tube from an erythrocyte-derived signal that originates in the circulation is therefore a real analytical problem, not a hypothetical one.

Public proteomic repositories make such questions addressable without new sample collection [27,28], provided that data reuse meets its own methodological requirements [29]. In a plasma proteomic study of men with and without OSA, severe OSA was reported to show an oxidative-stress signature [30]. Several of the proteins behind that signature are erythrocyte quality markers in the panel of Geyer et al. [7]; the original report interpreted them biologically and did not measure them against a quality marker panel [30]. What this analysis adds is a test of that signature against an external, cell-count-calibrated definition of erythrocyte-associated content, applied to the same data. We therefore performed a secondary analysis of the publicly deposited data of that study, with a second public OSA dataset for directional comparison. The primary objective was to determine whether the reported oxidative-stress enrichment can be attributed to erythrocyte-associated proteins. The null hypothesis was that the erythrocyte quality marker index does not differ between non-OSA, mild OSA and severe OSA participants, and that the reported enrichment persists when erythrocyte panel proteins are excluded from or accounted for in the analysis. Secondary objectives were to describe the differential signal that remains after accounting for erythrocyte-associated content, to compare the discrimination achieved by the erythrocyte index alone with that of the full proteome, and to report platelet and coagulation indices as comparison indices; the analysis of the second dataset was exploratory.

## 2. Materials and Methods

### 2.1. Data Source

This study is a secondary analysis of a publicly available plasma proteomic dataset. The raw data are deposited in the ProteomeXchange Consortium under accession PXD073409 and were originally reported by Müller et al. [30]. The source cohort was a single-centre, prospective, observational study of male participants; the proteomic subset comprised 45 individuals stratified by polysomnography into non-OSA (apnoea–hypopnoea index < 5, *n* = 15), mild OSA (5–15, *n* = 15) and severe OSA (>30, *n* = 15). Plasma was collected into EDTA tubes, prepared with the autoSP3 protocol, and acquired on an Orbitrap Exploris 480 in data-independent acquisition mode; proteins were identified and quantified in Spectronaut with cross-run normalisation enabled.

We used the protein-group-level quantification matrix (Dataset.xlsx, Supplementary Materials). Of 492 quantified protein groups, the 422 detected in at least 70% of samples were retained for analysis, and the 420 with at least 10 valid values in each compared group were tested. The 70% threshold was chosen as a compromise between retaining proteins and avoiding group estimates based on there being few observations in groups of 15; varying it to 50% and 80% did not change the results (Section 3.6). No clinical variables other than severity group were available to us; age, body mass index, apnoea–hypopnoea index and comorbidity data were not accessible. The original analysis used age-adjusted linear models and excluded proteins with any missing value [30]; neither was possible here, so our differential models are unadjusted for clinical covariates and retain proteins detected in at least 70% of samples.

### 2.2. Quality Marker Panels and Indices

Sample quality was assessed using the three quality marker panels defined by Geyer et al. [7]. These panels were derived from deep reference proteomes of erythrocytes, platelets and platelet-free plasma obtained from 20 healthy donors and were calibrated by serially diluting erythrocyte and platelet pools into platelet-free plasma with parallel cell counting; the resulting indices scale linearly with contaminating cell number (R = 0.98 and 0.99, respectively). Panel membership was taken from Table EV2 of that publication. NIF3L1 (erythrocyte panel) and F13A1 (platelet panel), which Geyer et al. excluded, were likewise excluded here. F13A1 is also a member of the coagulation panel and was retained there. Each protein group was represented by its first listed gene symbol, and a panel entry listing several symbols (for example ACTB;ACTG1) was counted as quantified when any of them matched.

Following the original definition, each quality marker index was computed as the summed intensity of the panel proteins divided by the summed intensity of all quantified proteins, using linear (untransformed) intensities. The coagulation index was computed from plasma-elevated members only, that is, those with a positive plasma–serum difference (higher in plasma than in serum) in the source publication [7]. The platelet and coagulation panels were used as comparison indices, allowing us to distinguish a finding specific to erythrocyte-associated protein content from one reflecting general sample degradation.

Of the 29 erythrocyte panel entries, corresponding to 36 gene symbols, 15 were quantified among the 492 protein groups of this dataset: ACTB, ALDOA, BLVRB, CA1, CA2, CAT, GAPDH, HBA1, HBB, HBD, LDHB, PRDX2, PRDX6, SLC4A1 and UBB. Indices were computed from all 15 using the full linear matrix. Three of them (ACTB, CA2, PRDX6) fell below the 70% detection threshold, so 12 panel members are present in the 422-protein analysis set and 11 met the valid-value requirement for testing, leaving 409 non-panel proteins in the adjusted analyses. Low-abundance panel members such as SPTA1, ANK1, PGK1 and TPI1 were not detected, consistent with the limited proteome depth of the source dataset.

### 2.3. Statistical Analysis

#### 2.3.1. Primary Analyses

Group differences in quality marker indices were assessed by one-way analysis of variance on log-transformed indices and by the Kruskal–Wallis test, differences between pairs of groups as ratios of geometric means with 95% confidence intervals from 4000 bootstrap resamples, and the association with ordinally coded group by Spearman correlation. Individual samples were screened with a within-dataset rule (mean + 2 SD), following Geyer et al. [7]; as the threshold is computed from the same samples it is descriptive rather than an independent reference.

Differential abundance analysis compared severe OSA with non-OSA using the criteria of the original publication: at least 10 valid values per group, Benjamini–Hochberg false discovery rate (FDR) control, with adjusted *p* values reported as q values, and thresholds of FDR < 0.25 with |log_2_ fold change| ≥ 0.5. Intensities were log_2_-transformed, and group differences were estimated by ordinary least squares as differences in mean log_2_ intensity. FDR < 0.05 was treated as the primary threshold; FDR < 0.25 with the fold-change filter, the rule of the original report, was treated as exploratory and was used to form the lists for over-representation analysis so that the original analysis could be reproduced. To account for variation in the erythrocyte quality marker index, its log_2_-transformed value was included as a covariate in an ordinary least squares model; panel members were excluded from this analysis to avoid circularity. In adjusted models, the group coefficient is reported as the adjusted log_2_ difference; fold-change filters were always applied to the unadjusted difference. Each protein was modelled on its complete observations, without imputation.

Over-representation analysis used one-sided Fisher exact tests with Benjamini–Hochberg correction, taking all tested proteins as background, since restricting the background to the observed proteome is essential when the measured space is a small and non-random subset of the proteome [31]. Gene sets were retrieved through gseapy (version 1.3.1) from the Enrichr libraries GO_Biological_Process_2023, GO_Molecular_Function_2023, and KEGG_2021_Human and Reactome_2022, downloaded on 28 August 2026 and again on 10 September 2026 with identical set sizes; the original report used MSigDB GO:BP, GO:MF and KEGG collections through clusterProfiler [30], so Reactome is an addition here. Sets with fewer than 5 or more than 500 annotated members were excluded before the background was restricted to the quantified proteome, so the overlap and set sizes reported in Table 1 refer to the quantified background and are smaller than the library sizes used for filtering. Enrichment statistics were computed locally so that the background could be controlled. Terms with fewer than two contributing proteins were not tested and were assigned *p* = 1, and the Benjamini–Hochberg correction was applied across all terms retained after filtering (906 for the 420-protein background and 855 for the 409-protein background).

To separate the effect of excluding panel members from the effect of covariate adjustment, differential analysis and over-representation were repeated under four scenarios: (A) all proteins, unadjusted; (B) panel members excluded, unadjusted; (C) panel members excluded, index-adjusted; and (D) all proteins, index-adjusted. In the last scenario, each panel member is adjusted for an index to which it contributes. It was therefore repeated with a leave-one-protein-out index for each panel member (scenario D1) because the three haemoglobin chains are co-expressed subunits that together contribute 98% of panel intensity, with an index excluding all three chains as the covariate for each haemoglobin chain (scenario D2); all other proteins were adjusted for the full index, as in scenario D.

#### 2.3.2. Secondary Analyses

Classification performance was evaluated by repeated stratified five-fold cross-validation. Median imputation, standardisation and L2-penalised logistic regression were applied within a pipeline fitted on training folds only, so that imputation medians and scaling parameters were never learned from held-out samples. No hyperparameter search was performed: C was prespecified at 0.1, a conventional value for strongly regularised high-dimensional problems, chosen because the feature-to-sample ratio (422 to 30) makes tuning on these data unstable; C = 0.01 and C = 1 were examined as a sensitivity analysis. Because the value was not selected on this dataset, no selection optimism arises from tuning; conversely, no attempt was made to optimise discrimination, so the reported AUROC values are not the maximum obtainable. Out-of-fold predicted probabilities were collected for every participant in each repeat and averaged across 50 random partitions to give one score per participant, from which AUROC, average precision, sensitivity, specificity and balanced accuracy were computed; confidence intervals were obtained from 2000 bootstrap resamples. Four feature sets were compared: all proteins, panel members excluded, panel members only, and the erythrocyte index as a single predictor. Because these models are fitted on the same participants, they were compared by paired differences in AUROC; participants (not cross-validation folds) were resampled 4000 times to obtain confidence intervals for each difference.

Two sensitivity analyses addressed index construction. First, the index was recomputed under three panel definitions: the full panel, the haemoglobin chains alone, and the non-haemoglobin members. Second, because panel members contribute to the index against which they are then correlated, a leave-one-protein-out index was computed for each member by removing it from both the numerator and the denominator, and an index excluding all three haemoglobin chains was computed for the haemoglobin chains themselves. The 70% detection threshold was varied to 50% and 80% to confirm that it does not drive the results.

To assess ordinal structure without in-sample optimism, the cross-validation described above was run on the non-OSA and severe OSA groups while the mild OSA group, which was never used in fitting, was scored by every fold model; averaging gives one out-of-fold probability for each of the 45 participants. Two additional contrasts were run with the same procedure: non-OSA versus all OSA participants, and mild versus severe OSA.

#### 2.3.3. Software, Reproducibility and Use of Generative Artificial Intelligence

All analyses were performed in Python (versions 3.11 to 3.13; package versions are listed in the repository) using pandas, numpy, scipy, statsmodels, scikit-learn, gseapy and matplotlib. All random seeds are fixed, so the analysis is deterministic given the same input files and package versions. The analysis code, quality marker definitions, gene-set term tables and derived tables are publicly available (see Data Availability Statement).

Generative artificial intelligence was used for code support and language support. Claude Opus 5 and Claude Opus 5.5 (Anthropic, San Francisco, CA, USA) and ChatGPT 5 (OpenAI, San Francisco, CA, USA) assisted in writing and debugging the analysis code and in the English language editing of the manuscript. These tools were not used to generate or alter data. The authors made all analytical decisions, checked all outputs against the data and the published code, and interpreted the results.

### 2.4. Second Dataset Used for Directional Comparison (Exploratory)

A second publicly deposited plasma proteomic OSA dataset was retrieved from ProteomeXchange (PXD069503; primary repository iProX, IPX0013741000), generated at a single centre in Beijing and reported by Zhao et al. [32]. The source study enrolled 142 adults classified by apnoea–hypopnoea index as controls (<5 events/h, *n* = 50), mild-to-moderate OSA (5 to <30 events/h, *n* = 45) and severe OSA (≥30 events/h, *n* = 47). Patients were recruited through a sleep centre and diagnosed by polysomnography, whereas controls were recruited through a physical examination centre and screened with a type 3 home sleep apnoea test. Proteomic profiling was performed in a discovery cohort of 45 participants; subtracting the validation cohort (32 severe, 30 mild-to-moderate and 35 control participants) from these totals gives 15 participants per group. The deposited subproject comprises 45 samples (30 OSA, 15 controls) acquired on an Orbitrap Fusion Lumos with label-free quantification and includes the Proteome Discoverer protein-level output (1184 protein groups, 32.9% missing values; the same number of proteins as reported in the source study). The repository record and the raw file names identify the instrument as an Orbitrap Fusion Lumos, whereas the source study describes data-dependent acquisition on a Q Exactive with processing in MaxQuant; the deposited protein table was used as provided. The abundance columns label 30 samples as OSA and 15 as controls, and the 30 OSA samples occupy two consecutive blocks of 15 (F1–F15 and F16–F30), corresponding to the severe and mild-to-moderate groups of the discovery cohort. With this assignment, the decision rules of the source study (two-sided *t*-test, *p* < 0.05 and fold change ≥ 1.5 or ≤ 0.67) reproduce the pattern of its reported differential results, whereas the reverse assignment does not (Table S18): 127 increased and 135 decreased proteins for severe OSA versus controls (reported, 153 and 137), 111 and 37 for mild-to-moderate OSA versus controls (155 and 46), and 17 and 85 for severe versus mild-to-moderate OSA (22 and 89). Participant-level clinical variables were not deposited, and, unlike the primary cohort, the source study included both men and women. Because mild and moderate OSA are combined in the source study and patients and controls were recruited through different services, the dataset is used for directional comparison and not as an external validation cohort.

Two comparisons were made in this dataset. First, the three quality marker indices were computed exactly as in the primary dataset and compared across the three groups (Kruskal–Wallis test and Spearman correlation with ordinally coded groups), between pairs of groups (ratio of geometric means with a 95% confidence interval from 4000 bootstrap resamples, Welch *t*-test on the log scale, Mann–Whitney test and area under the receiver operating characteristic curve), and between all OSA and control samples; this asks whether erythrocyte-associated content is again higher in cases, in samples collected and processed at a different centre with a different platform. Second, the proteins that remained significant after index adjustment in the primary dataset were examined for directional concordance, reported as the number changing in the same direction, with the corresponding effect sizes compared by Spearman correlation and a 95% confidence interval from 4000 bootstrap resamples of proteins. The severe-OSA-versus-control contrast was used because it corresponds to the primary contrast, and the all-OSA-versus-control contrast is reported alongside. For differential abundance, protein groups detected in at least 70% of samples were retained (689 of 1184), and a protein was tested in a contrast when it had at least 10 valid values in each compared group, as in the primary analysis. Log_2_-transformed normalised abundances were modelled by ordinary least squares with group as the predictor, without imputation, and *p* values were adjusted by the Benjamini–Hochberg procedure within each contrast. Models were fitted with and without the log_2_ erythrocyte index as a covariate, and the all-OSA-versus-control model also with the log_2_ platelet index; members of the panel used for adjustment were excluded from the adjusted analyses, as in the primary dataset. Haemoglobin subunit alpha is annotated as HBA2 in this dataset and was matched to HBA1 accordingly.

### 2.5. Ethics

This work is a secondary analysis of publicly available, de-identified data and involved no new human participants. The original cohort study was conducted with institutional ethics approval and written informed consent from all participants, in accordance with the Declaration of Helsinki [30]. The source study of the second dataset was approved by the Ethics Committee of Beijing Chao-Yang Hospital, and all participants gave written informed consent [32].

## 3. Results

### 3.1. Quality Marker Indices Differ Between Clinical Groups

**Figure 1:**
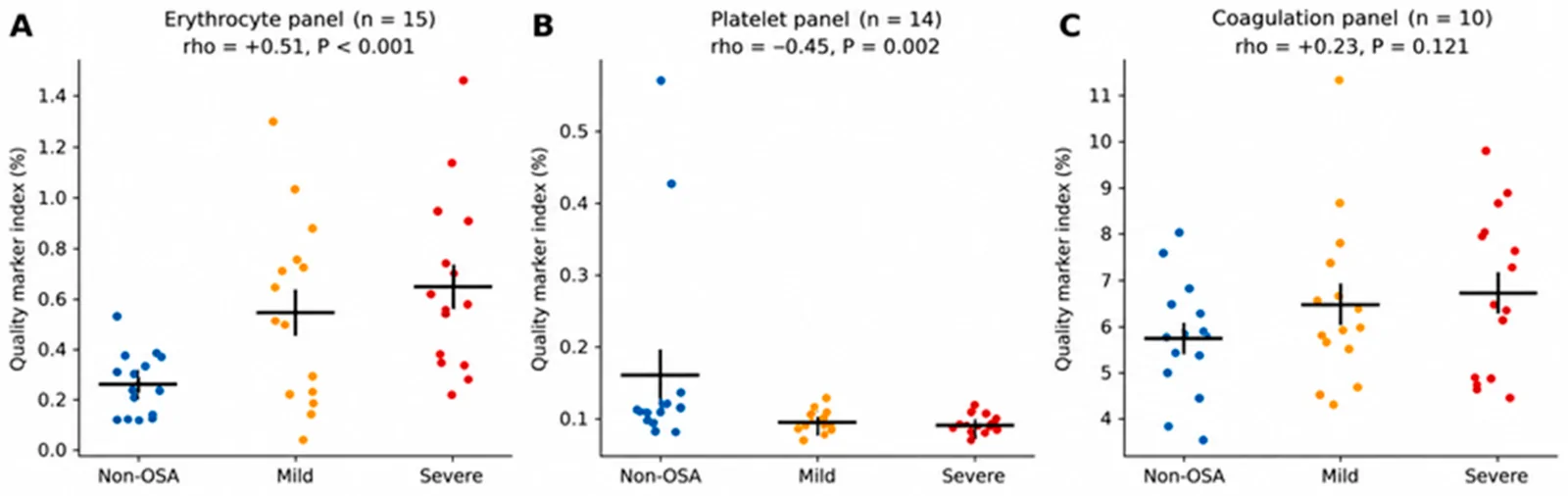
Quality marker indices by clinical group. (**A**) Erythrocyte, (**B**) platelet and (**C**) coagulation index. Each point is one participant; horizontal bars show group means with standard errors. Indices are the summed intensity of panel proteins divided by the summed intensity of all quantified proteins. rho is the Spearman correlation with ordinally coded group. Blue, orange, and red dots represent the Non-OSA, Mild, and Severe groups, respectively. Each dot represents an individual observation. Horizontal black bars indicate the group means, and vertical black bars indicate variability. Spearman’s correlation coefficients (rho) and corresponding *p*-values are shown above each panel.

Fifteen members of the erythrocyte panel, fourteen of the platelet panel and ten of the plasma-elevated coagulation panel were quantified in this dataset. The erythrocyte index was higher in mild and severe OSA than in non-OSA participants (non-OSA 0.264%, mild OSA 0.550%, severe OSA 0.655%; analysis of variance on the log scale *p* = 0.0029; Kruskal–Wallis *p* = 0.0024; Spearman rho = +0.51, *p* = 0.0003; Figure 1A). Relative to non-OSA participants, the ratio of geometric means was 1.74 (95% CI 1.04 to 2.80) for mild and 2.43 (1.68 to 3.47) for severe OSA, whereas the difference between severe and mild OSA was smaller and imprecisely estimated (1.40, 0.86 to 2.35; Table S1).

The group difference did not depend on how the index was constructed. Substituting a denominator restricted to non-panel proteins gave rho = +0.51, and two denominator-free definitions—the median z-score of panel members and their first principal component—gave rho = +0.45 (*p* = 0.0019) and rho = +0.47 (*p* = 0.0010). The four definitions agreed closely with one another (pairwise Spearman 0.97 to 1.00), and non-panel proteins showed no systematic negative association with the index (mean r = −0.005), as expected given that panel members account for well under 1% of total measured intensity.

The two comparison indices behaved differently. The platelet index was lower in both OSA groups (0.165%, 0.102%, 0.098%; rho = −0.45, *p* = 0.0018; severe-to-non-OSA ratio 0.71, 95% CI 0.52 to 0.91; Figure 1B), and this was not a consequence of the shared denominator, since an erythrocyte-independent denominator gave the same result (rho = −0.45, *p* = 0.0020). The coagulation index did not differ clearly between groups (rho = +0.23, *p* = 0.121; Figure 1C). The three indices did not move together; the correlation between the erythrocyte and platelet indices was modest (rho = −0.32). Different preanalytical processes can affect cell types differently, so this comparison is contextual rather than an exclusion test.

A within-dataset screening rule (mean + 2 SD, computed across all 45 samples) flagged two samples; because the threshold derives from the same dataset, it is not an independent quality reference. Few individual samples were therefore extreme, while the group means differed, indicating a shift distributed across groups rather than driven by outliers.

### 3.2. A Large Share of the Reported Differential List Consists of Panel Members

Applying the original study’s criteria (≥10 valid values per group, FDR < 0.25, |log_2_FC| ≥ 0.5) to the severe-versus-non-OSA contrast recovered 22 differentially abundant proteins, 13 increased in severe OSA (Table S2). Six of these 13 (46%) are members of the erythrocyte quality panel: CA1, HBA1, HBB, HBD, PRDX2 and SLC4A1. In the literature survey of Geyer et al. [7], 11% of studies reported two panel proteins and a further 11% reported three or more; six is well outside that distribution.

The original report described 24 differentially abundant proteins—11 increased and 13 decreased [30]. The difference from our 22 is expected, since the original used age-adjusted models and excluded proteins with any missing value, whereas age was unavailable to us and we retained proteins detected in at least 70% of samples. Effect sizes for the proteins central to this analysis were nonetheless identical to two decimal places (CRP log_2_FC 1.59; PRDX2 1.15; IGF1 −0.70), indicating that the difference lies in which proteins pass the filters rather than in the quantification.

SLC4A1 (band 3) is an integral erythrocyte membrane protein; its presence in plasma is consistent with erythrocyte lysis or with the release of red-cell-derived vesicles, and the two cannot be separated here.

The erythrocyte origin of several of these proteins was not overlooked in the original report. Its volcano plot assigns HBA1, HBB, HBD, CA1 and SLC4A1 to an “erythropoiesis footprint” category, and its enrichment figure lists oxygen carrier activity, haemoglobin binding and bicarbonate transport among the top terms [30]; PRDX2 was assigned to the oxidative-stress category. The interpretation offered there was biological. What the present analysis adds is an external reference: these proteins belong to a quality marker panel whose index, in its source study, scales linearly with contaminating cell number [7], the partly measured index differs between clinical groups in this dataset, and every term enriched in the unadjusted analysis consists of its members.

**Figure 2:**
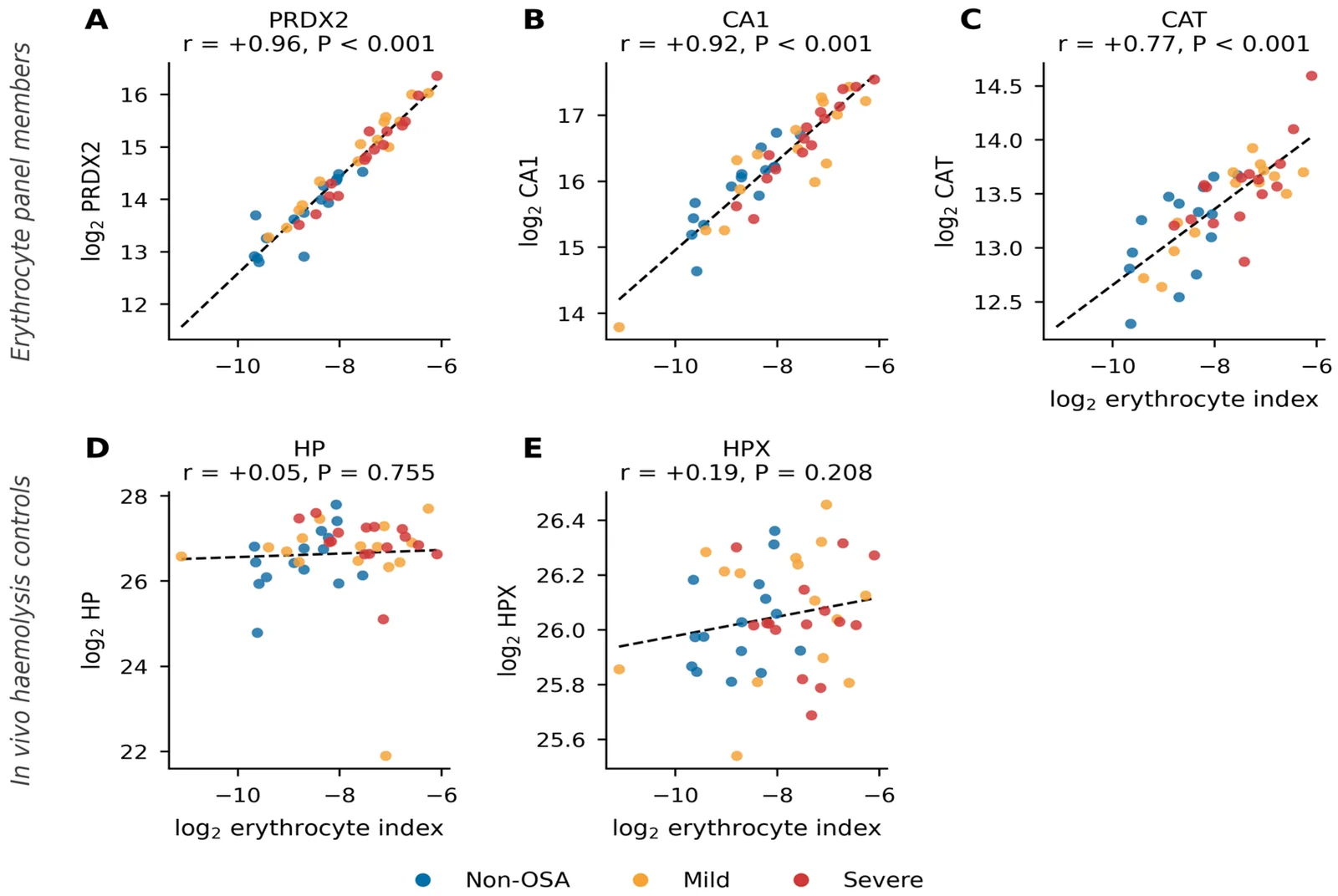
Panel members track the erythrocyte index, whereas haptoglobin does not. (**A**) Correlation between the erythrocyte index and peroxiredoxin 2 (PRDX2) (r = 0.96, *p* < 0.001). (**B**) Correlation between the erythrocyte index and carbonic anhydrase 1 (CA1) (r = 0.92, *p* < 0.001). (**C**) Correlation between the erythrocyte index and catalase (CAT) (r = 0.77, *p* < 0.001). (**D**) Correlation between the erythrocyte index and haptoglobin (HP) (r = 0.05, *p* = 0.755). (**E**) Correlation between the erythrocyte index and haemopexin (HPX) (r = 0.19, *p* = 0.208). Panels (**A**–**C**) show erythrocyte panel members, whereas panels (**D**,**E**) show HP and HPX as in vivo haemolysis controls. Each circle represents an individual sample. Blue, orange, and red circles indicate the Non-OSA, Mild, and Severe groups, respectively. Dashed lines represent least-squares linear regression fits. r denotes the Pearson correlation coefficient.

Individual panel members were tightly coupled to the index: PRDX2 correlated with the log-transformed erythrocyte index at r = +0.96, CA1 at r = +0.92, CAT at r = +0.77 and SLC4A1 at r = +0.54 (all *p* < 0.001; PRDX2, CA1 and CAT are shown in Figure 2A–C). Because each protein contributes to the index it is correlated with, these coefficients were recomputed with the protein removed from both the numerator and the denominator of the index. The values were essentially unchanged (PRDX2 +0.964, CA1 +0.922, CAT +0.773, SLC4A1 +0.535; Table S14), as expected given that these proteins account for 0.2%, 0.6%, 0.1% and less than 0.1% of total panel intensity. The haemoglobin chains are the exception, together contributing 98% of panel intensity; an index built without any haemoglobin chain still correlated with each of them at r = +0.72 to +0.74. For PRDX2, interpreted in the original report as an oxidative-stress marker, the correlation approaches identity, so its measured plasma abundance is largely determined by erythrocyte-associated content in this dataset.

### 3.3. Composition of the Enriched Terms

**Figure 3:**
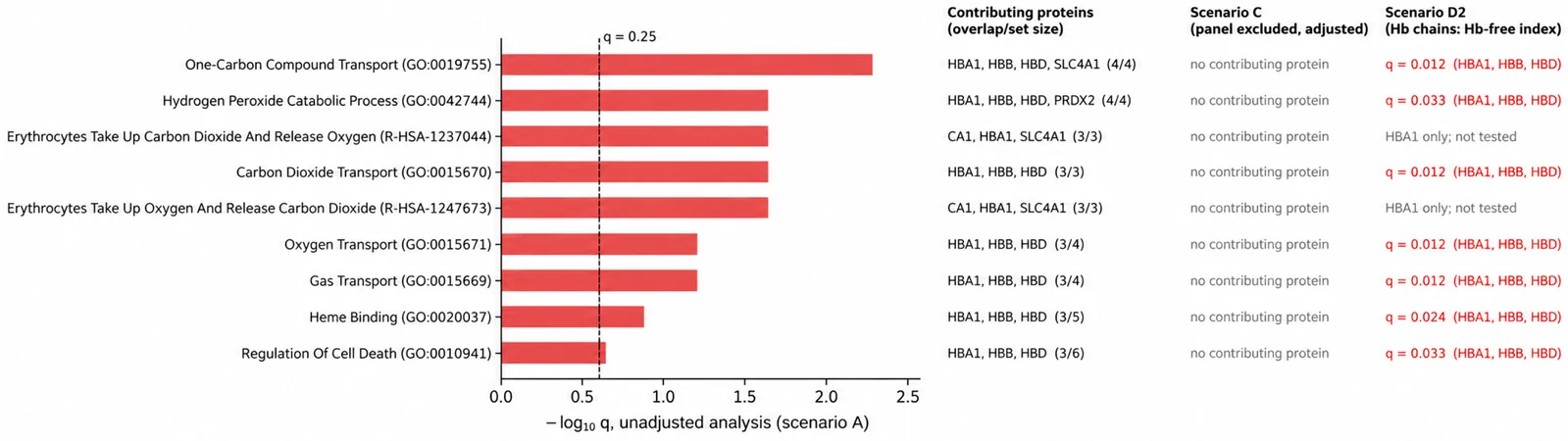
Composition of the enriched terms. The nine terms reaching q < 0.25 in the unadjusted analysis (scenario A), in order of significance, with the contributing proteins and overlap/set size. The right-hand columns give the result with panel members excluded, the index as covariate (scenario C; scenarios B, D and D1 gave the same result), and with each haemoglobin chain adjusted for an index excluding the haemoglobin chains (scenario D2). Terms with a single contributing protein were not tested. Gene sets were taken from GO Biological Process, GO Molecular Function, KEGG and Reactome, with the quantified proteome as background. The vertical dashed line indicates the adjusted significance threshold (q = 0.25). Red text denotes the adjusted q-values and contributing proteins identified in Scenario B using the Hb chains–free index.

Over-representation analysis against GO Biological Process, GO Molecular Function, KEGG and Reactome—with the quantified proteome as background—returned nine terms at q < 0.25 for the unadjusted differential list. Every one of them is an erythrocyte or a gas-transport or haem-related term, and each is driven by the same small group of proteins (Table 1; Figure 3; Table S6).

Hydrogen peroxide catabolic process is an antioxidant-defence term and could be interpreted as evidence of systemic oxidative stress. In this dataset its four contributing proteins are three haemoglobin chains and peroxiredoxin 2, all members of the erythrocyte panel. The same holds for response to hydrogen peroxide (HBA1, HBB; *p* = 0.024) and response to reactive oxygen species (HBA1, HBB; *p* = 0.035). Regulation of cell death is also driven only by haemoglobin chains. These annotations are biologically appropriate for erythrocyte proteins, but on their own they do not demonstrate altered oxidative activity in the plasma compartment.

Table 2 shows the differential and enrichment results under each scenario (Tables S8–S11). Excluding panel members, with or without adjustment (scenarios B and C), removed all nine terms; because every contributing protein is a panel member, this follows from the composition shown in Table 1 and is not independent evidence. With all proteins retained and adjusted for the full index (scenario D), no term reached q < 0.25, but in this scenario each panel member is adjusted for an index to which it contributes. A leave-one-protein-out index left this result unchanged (scenario D1). The haemoglobin chains, however, are co-expressed subunits, so the index that remains after one chain is removed is still dominated by the other two. When each chain was instead adjusted for an index excluding all three chains (scenario D2), HBA1, HBB and HBD remained elevated in severe OSA (q = 0.059, 0.071 and 0.157), whereas PRDX2, CA1 and SLC4A1 did not (q ≥ 0.44), and seven of the nine terms reached q < 0.25 again, including hydrogen peroxide catabolic process (q = 0.033), which now rested on the three haemoglobin chains alone (Table S11b,c). The haemoglobin chains therefore differed between groups by more than the other erythrocyte panel members did, in line with the smaller group difference in the non-haemoglobin index (Section 3.6). In every scenario, all proteins contributing to these terms were erythrocyte panel members. In scenario C, the strongest remaining signals were two broad regulatory terms driven by CRP and IGF1 (*p* = 0.027 and 0.035; q = 1.0; Tables S7 and S10), which are not interpretable at this significance level.

### 3.4. Discrimination Attributable to the Erythrocyte Index

**Figure 4:**
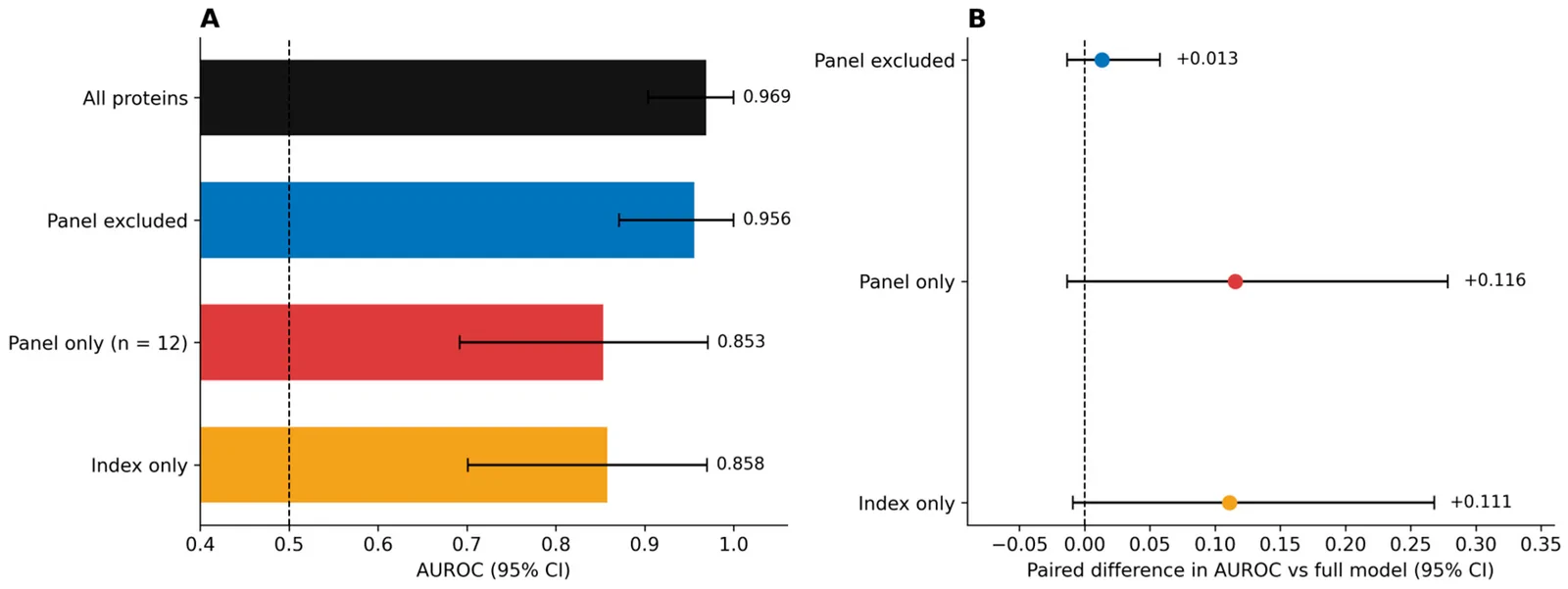
Discrimination of severe OSA from non-OSA. (**A**) AUROC for four feature sets, with 95% confidence intervals obtained from 4000 participant-level bootstrap resamples of the averaged out-of-fold predictions. Horizontal bars represent AUROC values, and horizontal error bars indicate the corresponding 95% confidence intervals. The dashed vertical line marks chance-level discrimination (AUROC = 0.50). (**B**) Paired differences in AUROC between the full model and each reduced feature set, with 95% confidence intervals obtained from the same bootstrap resampling procedure. Colored circles represent the estimated paired AUROC differences, and horizontal error bars indicate their 95% confidence intervals. The dashed vertical line indicates no difference in AUROC (ΔAUROC = 0). Models were fitted using repeated stratified five-fold cross-validation with 50 repeats.

Under repeated stratified five-fold cross-validation with 50 repeats, an L2-penalised logistic model using all 422 proteins separated severe OSA from non-OSA with an AUROC of 0.969 (95% CI 0.904 to 1.000). The erythrocyte index used as a single predictor reached 0.858 (0.701 to 0.970), and the 12 panel members present in the analysis set reached 0.853 (0.692 to 0.971). Excluding panel members left an AUROC of 0.956 (0.871 to 1.000) (Table 3; Figure 4; Table S5a,b). At the 0.5 probability threshold the full model achieved a sensitivity of 0.93 and a specificity of 0.87, and the index alone 0.73 and 0.73. Repeating the analysis with C = 0.01 and C = 1 changed the AUROC of each feature set by less than 0.025, and at every penalty strength the full and panel-excluded models remained above the erythrocyte index and the panel members alone (Table S5a).

The erythrocyte index alone showed high discrimination between the extreme groups, which is compatible with confounding by sample quality, although it does not on its own establish the origin or causal role of the signal. The paired differences favoured the full model over the index alone (+0.111, 95% CI −0.009 to +0.268) and over the panel members alone (+0.116, −0.013 to +0.278), but with 30 participants the intervals include both no difference and a substantial advantage, so the data cannot show whether the remaining proteins add discrimination beyond the index. Removing panel members from the full model changed discrimination little (+0.013, −0.013 to +0.058).

### 3.5. Signals Retained After Adjustment

**Figure 5:**
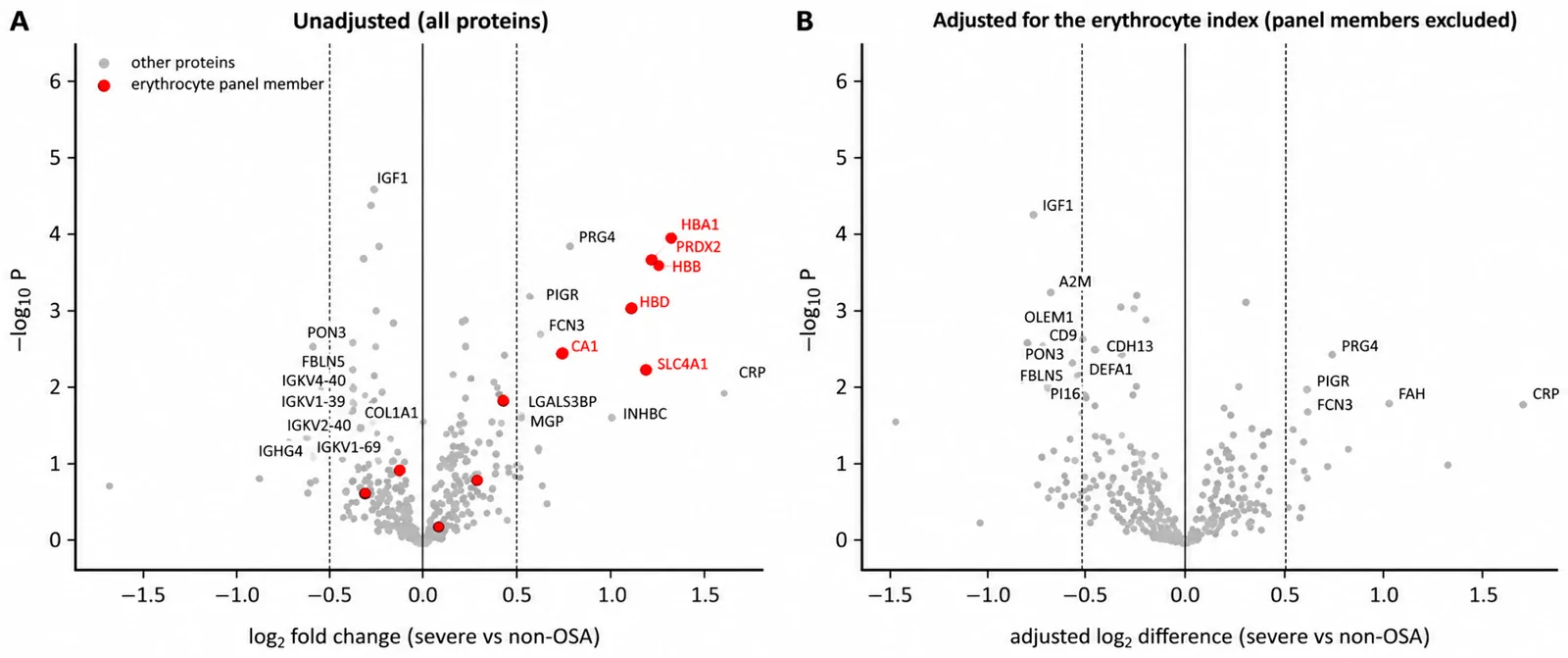
Differential abundance before and after adjustment. (**A**) Unadjusted, all proteins, and (**B**) adjusted for the erythrocyte index with panel members excluded; the horizontal axis in (**B**) is the adjusted difference. Red points are erythrocyte panel members; dotted vertical lines mark an absolute log_2_ difference of 0.5, and labelled proteins have q < 0.25 and an absolute difference of at least 0.5. The solid vertical lines indicate zero log_2_ fold change or adjusted log_2_ difference, while the dotted vertical lines indicate the predefined effect-size thresholds. Each point represents an individual protein. Red points in panel (**A**) represent erythrocyte panel members, whereas grey points represent other proteins. Selected proteins are labelled for interpretation.

Decision rules are reported separately here to avoid ambiguity. Across the 420 tested proteins, 16 reached q < 0.05 and 84 reached q < 0.25 without a fold-change filter; adding the |log_2_FC| ≥ 0.5 filter used in the original report gives the 22 proteins of Section 3.2. Restricting to the 409 non-panel proteins and adding the log-transformed erythrocyte index as a covariate reduced the count from 11 to 1 at q < 0.05 and from 73 to 30 at q < 0.25 (Table S3; Figure 5). We treat q < 0.05 as the primary threshold and q < 0.25 as exploratory, following the original report. At the exploratory threshold, 26 proteins that were significant before adjustment remained significant after it; at the primary threshold only IGF1 did (unadjusted log_2_FC −0.70, adjusted q = 0.016), followed at q ≤ 0.077 by A2M, SERPINC1, BCHE, CLEC3B, ALB, GC and SERPINA6. CRP retained its direction and magnitude. Four proteins (CHL1, FAH, DEFA1 and VASN) reached the exploratory threshold only after adjustment, and for 25 of the 30 proteins significant after adjustment the adjusted difference was larger than the unadjusted one (Table S4). Because the index differs between groups, adjusting for it can enlarge as well as reduce a group difference, and it can also remove disease-related variation that is correlated with the index.

### 3.6. Sensitivity Analyses and the Location of the Discriminating Signal

Recomputing the index from the three haemoglobin chains alone gave essentially the same group difference (2.52-fold, rho = +0.52, *p* = 0.00027), whereas the twelve non-haemoglobin panel members gave a weaker and non-significant one (1.35-fold, rho = +0.27, *p* = 0.077; Table S12a). The difference is therefore carried predominantly by the most abundant panel members. Varying the detection threshold from 70% to 50% left the analysis set and every count unchanged, and raising it to 80% left 411 proteins, the same index association (rho = +0.51) and the same adjusted result (1 protein at q < 0.05, 25 at q < 0.25; Table S12b), so the threshold does not drive the findings.

**Figure 6:**
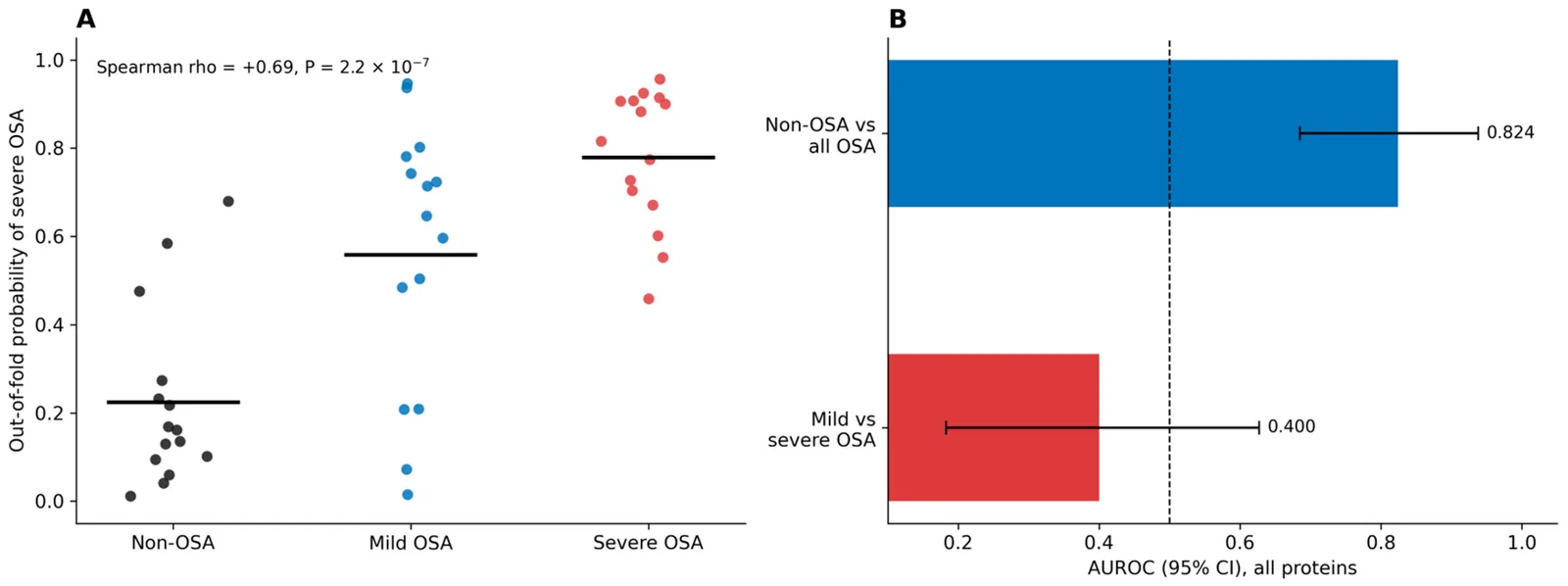
Ordinal position of the mild OSA group and the location of the discriminating signal. (**A**) Out-of-fold probability of severe OSA for all 45 participants. Models were trained on non-OSA and severe OSA; each participant was scored only by fold models that had not seen it, and the mild OSA group was never used in fitting. Black, blue, and red dots represent individual participants in the non-OSA, mild OSA, and severe OSA groups, respectively. Horizontal black lines indicate group means. Spearman’s correlation coefficient (rho) and the corresponding *p*-value are displayed above the panel. (**B**) AUROC with 95% confidence intervals for two contrasts using all proteins: non-OSA versus all OSA participants, and mild versus severe OSA. Blue and red horizontal bars represent the AUROC values for the respective contrasts, while horizontal error bars indicate their 95% confidence intervals. The dashed vertical line indicates chance-level discrimination (AUROC = 0.50).

Out-of-fold probabilities, obtained by training on non-OSA and severe OSA and scoring every participant only when held out, rose across groups (non-OSA 0.22 ± 0.20; mild OSA 0.56 ± 0.30; severe OSA 0.78 ± 0.16; Spearman rho = +0.69, *p* = 2.2 × 10^−7^; Figure 6A; Table S13a). The ordering is therefore reproduced without in-sample optimism, but the dispersion is large and the groups overlap substantially. Two further contrasts locate the signal. Non-OSA participants were separated from all OSA participants with an AUROC of 0.824 (95% CI 0.685 to 0.938), whereas mild OSA was not separated from severe OSA at all (0.400, 95% CI 0.183 to 0.627, all proteins; 0.556, 0.330 to 0.764, index alone; Figure 6B; Table S13b). In this dataset the proteome therefore separated participants with OSA from those without, but not mild from severe OSA; with 15 participants per group this does not establish whether the difference is confined to the presence of OSA or whether a difference between severity grades was too small to detect. The erythrocyte index behaved in the same way.

### 3.7. Quality Marker Content in the Second OSA Dataset (Exploratory)

**Figure 7:**
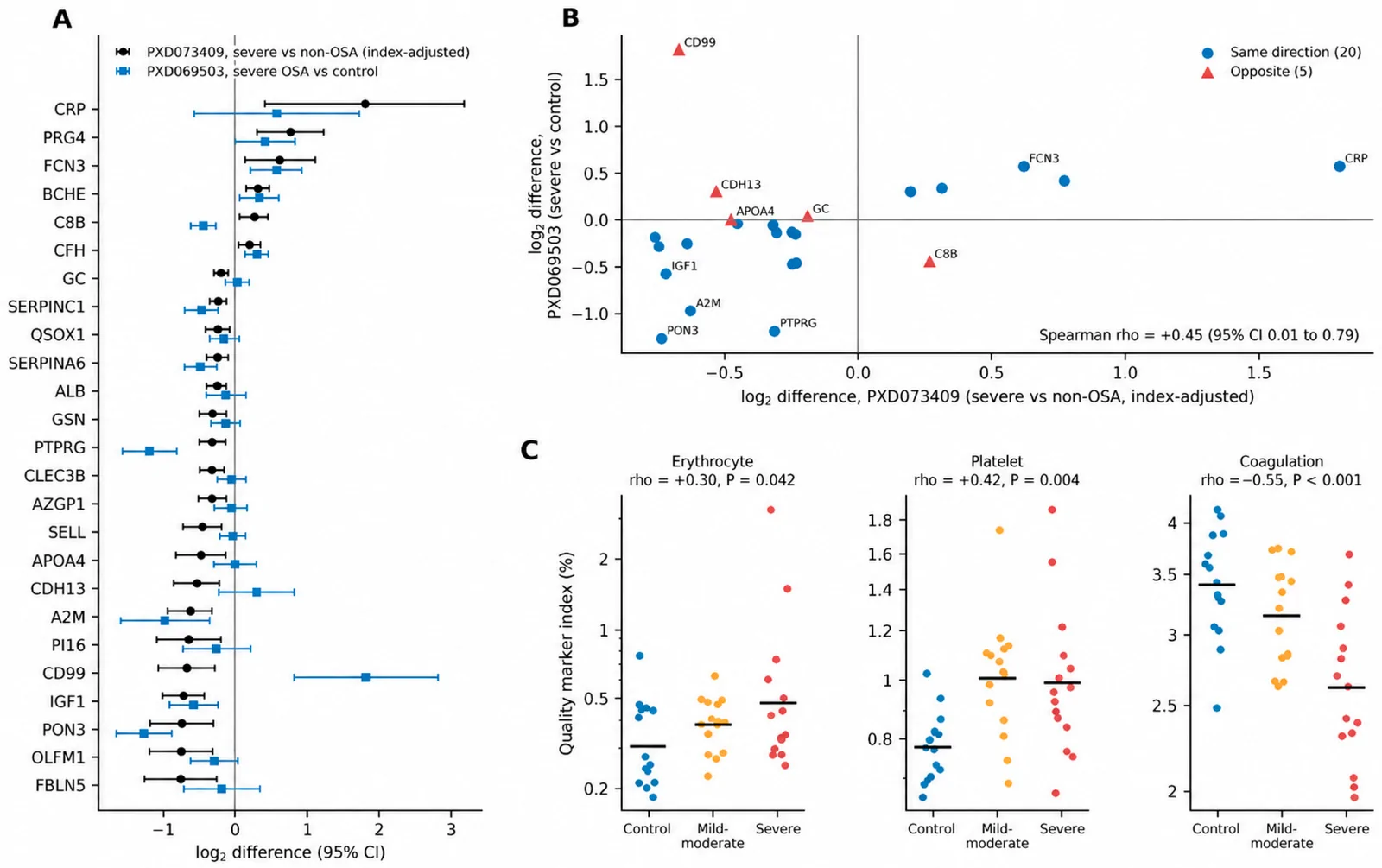
Quality marker content and directional concordance in the second dataset (PXD069503). (**A**) The 25 adjustment-persistent proteins of the primary analysis that were also quantified here, showing the index-adjusted difference between severe and non-OSA in PXD073409 and the difference between severe OSA and controls in PXD069503, with 95% confidence intervals. (**B**) The same effect sizes plotted against each other, with the Spearman correlation and a 95% confidence interval from 4000 bootstrap resamples of proteins. (**C**) Erythrocyte, platelet and coagulation quality marker indices in control, mild-to-moderate OSA and severe OSA samples; horizontal bars are geometric means, and rho is the Spearman correlation with ordinally coded groups. Note the logarithmic ordinate.

The same panels were applied to a second, independently generated OSA plasma proteomic dataset (PXD069503 [32]; 15 severe OSA, 15 mild-to-moderate OSA, 15 controls; Orbitrap Fusion Lumos, label-free), which is used for directional comparison only. Twenty-seven erythrocyte, twenty-eight platelet and twelve coagulation panel members were quantified. The erythrocyte index was 1.57-fold higher in all OSA than in controls (0.53% versus 0.34%; Welch *p* = 0.033; Mann–Whitney *p* = 0.031; AUROC 0.70), reproducing the direction observed in the primary dataset (Figure 7C) in a different laboratory, on a different instrument and with a different acquisition mode. Across the three groups the index was highest in severe OSA (means: controls 0.34%, mild-to-moderate 0.39%, severe 0.66%; Spearman rho = +0.30, *p* = 0.042; Kruskal–Wallis *p* = 0.092). Severe OSA exceeded controls (ratio of geometric means 1.55, 95% CI 1.06 to 2.35), whereas the ratios of mild-to-moderate OSA to controls (1.24, 0.95 to 1.55) and of severe to mild-to-moderate OSA (1.25, 0.90 to 1.88) were imprecisely estimated (Table S15).

The two comparison panels behaved differently from the primary dataset. The platelet index was 1.31-fold higher in OSA (*p* < 0.001; AUROC 0.83), whereas in the primary dataset it was lower in OSA, and the coagulation index was lower in OSA (0.85-fold; *p* = 0.0014). By group, the platelet index was raised to a similar extent in mild-to-moderate OSA (ratio to controls 1.29, 95% CI 1.14 to 1.46) and severe OSA (1.27, 1.10 to 1.48), whereas the coagulation index decreased with severity (Spearman rho = −0.55, *p* < 0.001; severe to mild-to-moderate OSA 0.83, 0.74 to 0.93). Platelet panel members occupied much of the top of the differential list: of the 97 proteins increased in OSA at q < 0.05 (Table S16), 20 are platelet panel members, including YWHAZ, TMSB4X, VCL, PFN1, CFL1 and TAGLN2 with log_2_ fold changes between 1.9 and 3.7. In the severe-OSA-versus-control contrast, 95 proteins were increased at q < 0.05, 19 of them platelet panel members. In this dataset, therefore, it is platelet-associated rather than erythrocyte-associated content that aligns most strongly with the clinical grouping. Adjusting for the platelet index, with platelet panel members excluded, left 117 proteins at q < 0.05, so the signal there is not reducible to a single index.

Of the 26 proteins that remained significant after index adjustment in the primary dataset, 25 were quantified here. In the severe-OSA-versus-control contrast, which corresponds to the primary comparison, 20 of the 25 changed in the same direction and the two sets of effect sizes were positively correlated (Spearman rho = +0.45, 95% CI 0.01 to 0.79; Figure 7A,B); in the all-OSA-versus-control contrast the corresponding values were 19 of 25 and rho = +0.39 (95% CI −0.04 to 0.71). Eight were concordant and independently significant in severe OSA versus controls (IGF1, A2M, SERPINC1, SERPINA6, PTPRG, PON3, CFH and FCN3, all q < 0.05), while CD99 and C8B were significant in the opposite direction (Table S17). Because plasma proteins are correlated, the 26 were selected in the primary dataset, and the two cohorts differ in design, this comparison is descriptive, and no significance test is attached to the count.

## 4. Discussion

We reanalysed a public plasma proteomic dataset from an OSA cohort using quality marker panels defined and calibrated in an independent study. Erythrocyte-associated protein content was higher in OSA than in non-OSA participants; six of the thirteen proteins that were recovered as elevated in severe OSA belong to the erythrocyte panel; every pathway term enriched in the unadjusted list consisted exclusively of panel members; PRDX2 was accounted for by erythrocyte-associated content, whereas the haemoglobin chains remained elevated after adjustment for the other panel members; and the erythrocyte index alone discriminated the extreme groups with an AUROC of 0.858. A substantial part of the differential signal nonetheless persisted after adjustment, and the erythrocyte index was again higher in cases than controls in a second, independently generated OSA dataset.

### 4.1. Interpretation of the Enrichment Result

Hydrogen peroxide catabolic process reached q = 0.023 in a cohort defined by intermittent hypoxia, where an antioxidant signal is expected. Its four contributing proteins were three haemoglobin chains and peroxiredoxin 2. Haemoglobin carries peroxide- and oxygen-related annotations because of its function inside the erythrocyte, and peroxiredoxin 2 is among the most abundant erythrocyte proteins; their joint appearance in plasma indexes red-cell content. These annotations are appropriate at the level of the individual protein but do not by themselves indicate altered oxidative activity in plasma. When each haemoglobin chain was adjusted for an index that excluded the haemoglobin chains, PRDX2 no longer differed between groups and the term rested on the three chains alone, which carry the annotation because of the peroxidase activity of haemoglobin itself rather than that of an antioxidant enzyme. Geyer et al. [7] anticipated this, listing haemoglobin subunits, carbonic anhydrases, peroxiredoxin 2, superoxide dismutase and aldolase A as oxidation-annotated leakage proteins and citing a type 1 diabetes study in which the same attribution appears to have been made. The original OSA report did recognise an erythrocyte component, labelling several of these proteins as an erythropoiesis footprint [30]; the distinction we draw is between attributing them to erythropoiesis as biology and measuring them against a panel that was calibrated to contaminating cell number in its source study. In the present dataset the erythrocyte index also aligned with the clinical grouping rather than varying at random.

### 4.2. Preanalytical and In Vivo Origin

These data do not distinguish preanalytical erythrocyte carry-over from an in vivo erythrocyte-related signal, and both may contribute. Two observations are consistent with a preanalytical component. Haptoglobin was not depleted overall: in intravascular haemolysis, free haemoglobin binds haptoglobin and the complex is cleared, so a negative association between haptoglobin and the erythrocyte index would be expected, whereas we observed r = +0.05 (*p* = 0.755) across all samples (Figure 2D); haemopexin was likewise unrelated (r = +0.19, *p* = 0.208; Figure 2E). Separately, EDTA plasma—used in the source cohort—has been shown to carry higher erythrocyte-specific protein levels than lithium heparin or citrate plasma, with additional effects from vacuum sampling and gel-containing tubes [7]. Haptoglobin is also an acute-phase protein, however, so increased synthesis could offset consumption and mask a genuine haemolytic component, and the association is not uniform across groups: within the severe OSA group it was negative (rho = −0.45, *p* = 0.095), the direction expected under consumption.

Several observations support a possible in vivo contribution. Haptoglobin-related protein, which also binds haemoglobin, was inversely associated with the index across all samples (rho = −0.47, *p* = 0.001), and soluble CD163, the haemoglobin–haptoglobin scavenger receptor shed on macrophage activation, was positively associated (r = +0.33, *p* = 0.029); neither is specific, but both point in the direction of haemoglobin handling in vivo. Severity-dependent changes in the erythrocyte proteome have been reported in OSA [20]; erythrocyte dysfunction with excessive oxidative stress has been described as an early pathogenic mechanism [21]; haemoglobin concentration and red cell distribution width track severity and outcome [22,23,24,25]; and exhaled carbon monoxide has been studied as a haemolysis surrogate in OSA [26]. SLC4A1 in plasma is compatible with either lysis or vesicle release. The erythrocyte index was also elevated in cases in the second dataset (Section 3.7), which was collected and processed at a different centre on a different platform. This argues against an artefact confined to one laboratory, but not against a handling difference between groups: in that study, patients were recruited through a sleep centre and controls through a physical examination centre, and blood collection tubes and time to processing are not reported [32], so the conditions of blood collection may also have differed between groups there. We did not have access to preanalytical records—time from draw to centrifugation, centrifugation protocol, and tube lot—and could not test the handling hypothesis directly. The analysis identifies erythrocyte-associated protein content but does not determine its origin; interpreting these proteins as a systemic oxidative-stress programme would require independent evidence.

### 4.3. Relation to the Wider Literature

Systematic evaluations have shown that preanalytical factors significantly affect the plasma proteome in most studies designed to detect them [8]; that enrichment-based workflows are susceptible to cellular carry-over and can inflate apparent protein counts [5,6,15]; that variation between commercial standard operating procedures alters recovery [11]; and that affinity platforms are similarly affected by haemolysis [16]. In this dataset, the erythrocyte axis aligned with the clinical grouping, so a sample-quality signal may be read as disease biology rather than as additional noise. Oxidative stress is an established component of OSA pathophysiology [18,19], and this prior biological plausibility may increase the risk of overinterpretation.

### 4.4. Signals Persisting After Adjustment

The proteins that remained significant after adjustment are not erythrocyte-associated. IGF1, the strongest, was also the most significantly reduced protein in the original report; reduced IGF1 in severe OSA is compatible with somatotropic axis suppression in sleep-disordered breathing [1], but is also sensitive to adiposity and hepatic synthetic function, neither of which could be adjusted for. SERPINC1, A2M, CLEC3B, ALB and GC are largely hepatically synthesised proteins whose coordinated reduction is compatible with a shift in synthetic function or with residual confounding by body mass index. BCHE, also of hepatic origin, was increased rather than reduced. We regard this list as hypothesis-generating.

### 4.5. Reported Discrimination in Small Proteomic Cohorts

AUROC values in small cohorts should be read with the erythrocyte axis in view. Here, the index alone reached 0.858 and the full proteome 0.969, a paired difference of +0.111 whose confidence interval (−0.009 to +0.268) includes zero, while removing panel members left discrimination essentially unchanged. On these data a single index of erythrocyte content reached an AUROC of 0.858, and the cohort is too small to show whether 422 proteins do better; a high AUROC in a cohort of this size therefore says little about disease-specific information. Cross-validation protects against overfitting but not against a factor present in both training and test folds. Sample size requirements for reliable classification in omics data are considerably larger than are commonly used [33], and external validation in an independent cohort remains the only adequate test.

### 4.6. Directional Comparison with the Second Dataset

The second dataset (Section 3.7) is consistent with two of the present findings and qualifies a third. Erythrocyte-associated content was again higher in cases than controls—in samples generated independently—which argues against a signal confined to one laboratory, although recruitment pathway and clinical group coincided in that study (Section 4.2). Across severity groups, the index was highest in severe OSA and close to control values in mild-to-moderate OSA, whereas in the primary dataset, mild OSA was already close to severe OSA; in both datasets the difference between the two OSA groups was imprecisely estimated, so neither supports a graded relation with severity. The proteins persisting after adjustment were largely concordant in direction (20 of 25 in severe OSA versus controls), though less uniformly than a selected subset would suggest. The qualification concerns which panel dominates: in the second dataset the platelet index discriminated cases from controls better than the erythrocyte index, and platelet panel members occupied much of the top of the differential list. The platelet index was raised to a similar extent in both OSA groups, and the coagulation index decreased with severity, a pattern not seen in the primary dataset that these data cannot attribute either to sample handling or to in vivo processes. The two datasets therefore agree that quality marker content aligns with the clinical grouping while differing in which cellular compartment carries it, which is what would be expected if collection and processing conditions differ between centres. This supports reporting all three indices rather than any single one. The source article interpreted its proteomic findings as activation of glycolysis, platelets and the actin cytoskeleton in OSA [32]. Several of the proteins underlying that interpretation are quality panel members: TPI1, ALDOA, PGK1 and LDHB belong to the erythrocyte panel; ITGA2B, ITGB3, TLN1, FERMT3, PKM and ACTN1 to the platelet panel; and GAPDH and ACTB to both. This does not show that those findings are artefactual, and in that study, PKM—among other proteins—gave consistent results by immunoassay in a separate cohort. It does show that the situation examined here for the primary dataset, in which quality panel proteins enter a biological interpretation without reference to sample quality markers, is not confined to one study.

### 4.7. Limitations

The distinction between preanalytical and in vivo origin cannot be made with these data, as discussed above. The group difference in the index is carried mainly by the haemoglobin chains; excluding them leaves a weaker, non-significant trend among the remaining panel members (1.35-fold, *p* = 0.077). Limited proteome depth is the most likely explanation, since the dataset comprises 492 protein groups and low-abundance panel members such as SPTA1, ANK1, PGK1 and TPI1 were not detected, but a haemoglobin-specific phenomenon cannot be excluded. The same pattern limits adjustment: an index built from the non-haemoglobin members, which are fewer and less abundant, did not account for the excess of the haemoglobin chains (scenario D2), and adjustment for an imprecisely measured covariate leaves residual confounding. Only 15 of the 29 panel entries were quantified and 12 survived the detection filter, so the index is a partial implementation of a panel calibrated in its source study, and that calibration cannot be assumed to carry over to the measured subset. The proteome separated OSA from non-OSA participants but not mild from severe OSA, so the index should be read as associated with the clinical grouping rather than as a graded marker of severity. The cohort is small (*n* = 45), single-centre and male-only; OSA presents differently in women and sex-specific screening models are an active area of work [3], so these findings should not be extrapolated across sexes. We had no access to AHI, BMI, age or comorbidity data, and adjustment for adiposity in particular was not possible; this limits attribution of the differential signal—and of the erythrocyte index difference itself—to OSA severity rather than to correlated clinical features. Confidence intervals for AUROC differences were obtained by resampling participants from averaged cross-validated predictions; this reflects sampling variability but not the full uncertainty of model fitting, and is therefore likely to be optimistic. Over-representation analysis used the quantified proteome as background, which is appropriate but leaves small overlaps (three to four proteins for the significant terms), so individual *p* values rest on few proteins. The classification analyses are exploratory: with 30 participants in the main contrast and more than 400 candidate features, they indicate how much of the separation the erythrocyte index carries, not how a classifier would perform in new samples. The second dataset cannot test the central question: it was generated on a different platform with data-dependent acquisition; its severity groups rest on the order of the deposited samples, checked against the published results (Table S18); mild and moderate OSA are combined; participant-level clinical data were not deposited; it includes both sexes; and in the full source cohort, body mass index and the prevalence of hypertension were higher in severe OSA than in controls [32]. It is used for directional comparison only.

## 5. Conclusions

In a public plasma proteomic dataset from men with and without OSA, erythrocyte-associated protein content was higher in OSA, and the oxidative-stress enrichment reported for severe OSA consisted entirely of erythrocyte quality panel proteins. PRDX2 tracked erythrocyte content, and the terms that remained after adjustment rested on the haemoglobin chains alone, so on these data the enrichment is not evidence of altered oxidative activity in plasma. Part of the differential signal persisted after adjustment, and erythrocyte- and platelet-associated content was also higher in OSA in a second dataset. Whether the erythrocyte-associated content arises before or after venepuncture cannot be determined, and the analyses were not adjusted for clinical covariates. Plasma proteomic biomarker studies should report erythrocyte, platelet and coagulation quality marker indices alongside their differential results.

## Figures and Tables

**Table 1:** Enriched terms in the unadjusted severe-versus-non-OSA list. All contributing proteins are members of the erythrocyte quality panel.

Term	Set Size	Overlap	*p*	q	Contributing Proteins
One-Carbon Compound Transport (GO:0019755)	4	4	6.0 × 10^−6^	0.005	HBA1, HBB, HBD, SLC4A1
Hydrogen Peroxide Catabolic Process (GO:0042744)	6	4	8.0 × 10^−5^	0.023	HBA1, HBB, HBD, PRDX2
Erythrocytes Take Up Oxygen And Release Carbon Dioxide (R-HSA-1247673)	3	3	1.3 × 10^−4^	0.023	CA1, HBA1, SLC4A1
Erythrocytes Take Up Carbon Dioxide And Release Oxygen (R-HSA-1237044)	3	3	1.3 × 10^−4^	0.023	CA1, HBA1, SLC4A1
Carbon Dioxide Transport (GO:0015670)	3	3	1.3 × 10^−4^	0.023	HBA1, HBB, HBD
Oxygen Transport (GO:0015671)	4	3	4.9 × 10^−4^	0.063	HBA1, HBB, HBD
Gas Transport (GO:0015669)	4	3	4.9 × 10^−4^	0.063	HBA1, HBB, HBD
Heme Binding (GO:0020037)	5	3	1.2 × 10^−3^	0.133	HBA1, HBB, HBD
Regulation Of Cell Death (GO:0010941)	6	3	2.3 × 10^−3^	0.228	HBA1, HBB, HBD

**Table 2:** Differential abundance and enrichment under four analysis scenarios and two variants of scenario D. Overlap is the number of the nine terms enriched in the unadjusted analysis that retain any contributing protein. Counts in parentheses add the |log_2_ fold change| ≥ 0.5 filter used in the original report. In D1, each erythrocyte panel member is adjusted for an index from which it has been removed; in D2, each haemoglobin chain is adjusted for an index from which all three chains have been removed. In D2, eight terms reached q < 0.25: seven of the nine and positive regulation of cell death (GO:0010942).

Scenario	Proteins Tested	q < 0.05	q < 0.25	Terms at q < 0.25	Overlap with the Nine Terms
A. All proteins, unadjusted	420	16	84 (22)	9	9
B. Panel members excluded, unadjusted	409	11	73 (15)	0	0
C. Panel members excluded, index-adjusted	409	1	30 (8)	0	0
D. All proteins, index-adjusted	420	1	26 (6)	0	0
D1. As D, leave-one-protein-out index	420	1	26 (6)	0	0
D2. As D1, haemoglobin chains adjusted for an index without haemoglobin chains	420	1	33 (11)	8	9 (HBA1, HBB, HBD; HBA1 only in two)

**Table 3:** Discrimination of severe OSA from non-OSA, with paired differences. Confidence intervals are from 4000 participant-level bootstrap resamples of the averaged cross-validated predictions.

Feature Set	AUROC (95% CI)	Difference vs. Full Model	95% CI of the Difference
All 422 proteins	0.969 (0.904–1.000)	–	–
Panel members excluded	0.956 (0.871–1.000)	+0.013	−0.013 to +0.058
Panel members only (12)	0.853 (0.692–0.971)	+0.116	−0.013 to +0.278
Erythrocyte index alone	0.858 (0.701–0.970)	+0.111	−0.009 to +0.268

## Data Availability

This study is a secondary analysis of publicly available data. The mass spectrometry proteomics data were generated by Müller et al. [30] and are available from the ProteomeXchange Consortium via the PRIDE partner repository under accession PXD073409. The second dataset, used for directional comparison, is available under accession PXD069503 (iProX IPX0013741000) [32]. The quality marker panels were taken from Table EV2 of Geyer et al. [7], whose underlying data are available under accession PXD011749. The analysed subsets of these three sources are provided together as Dataset.xlsx in the Supplementary Materials. The analysis code, quality marker definitions, gene-set term tables and derived tables are available at https://github.com/drhilal/OSA (accessed on 6 October 2026). No new primary data were generated.

## Supplementary Materials

The following supporting information can be downloaded at: https://www.mdpi.com/article/10.3390/jcm15208081/s1, Table S1: Quality marker indices by severity group, with ratios of geometric means and bootstrap intervals; Table S2: Unadjusted differential abundance; Table S3: Differential abundance adjusted for the erythrocyte index; Table S4: Side-by-side comparison of unadjusted and adjusted results with the status of each protein after adjustment; Table S5a: Classification results for four feature sets at three penalty strengths; Table S5b: Paired AUROC differences with bootstrap intervals; Table S6: Over-representation analysis before adjustment; Table S7: Over-representation analysis after adjustment; Tables S8–S11: Over-representation analysis under scenarios A–D of Table 2; Table S11b: Differential abundance under scenarios D1 and D2; Table S11c: Over-representation analysis under scenario D2; Table S12a: Index sensitivity to panel definition; Table S12b: Counts at detection thresholds of 50%, 70% and 80%; Table S13a: Per-participant out-of-fold scores; Table S13b: Non-OSA versus all-OSA and mild versus severe contrasts; Table S14: Leave-one-protein-out index correlations; Table S15: Quality marker indices in PXD069503 by group; Table S16: Differential abundance in PXD069503; Table S17: Directional concordance between PXD073409 and PXD069503; Table S18: Differential protein counts in the source study of PXD069503 compared with the group assignment used here. Dataset.xlsx contains the analysed data: sheet OSA_plasma_proteome (PXD073409 quantification matrix, 45 samples, 492 protein groups), sheet OSA_plasma_proteome_2 (PXD069503 Proteome Discoverer output, 1184 protein groups; columns F1–F15 severe OSA, F16–F30 mild-to-moderate OSA, F31–F45 controls) and sheet Geyer_quality_panels (Table EV2 of Geyer et al. [7]). The source articles are open access under CC BY 4.0 and the material is reproduced with attribution.
